# Veterinary Register (*Continued*)

**Published:** 1902-04

**Authors:** 


### VETERINARY REGISTER.

[See Editorial, Journal of Comparative Medicine and Veterinary Archives, July, 1901, page 438.]

[In the following list the names and data printed without comment are those furnished to us by postal card with the signature of the individual.

Those with an asterisk [*] are those to whom we have sent, by United States mail, an envelope with prepaid postage and a request for return if not delivered in ten days; as the envelopes have not been returned, we presume that the letter has been delivered, that the individual in question exists, but that he has neglected to return the enclosed postal card with the information requested.—Ed. Journal.]

### PENNSYLVANIA.

(Continued from page 200.)

| *Name.* | *Street.* | *Town.* | *College, Graduated.* | *County, Registered.* |
|---|---|---|---|---|
| Seitz, Elmer E. | ...... | Glen Rock | National Vet., 1896 | York, 1896 |
| Seitz, Jacob H. | ...... | Manor Station | * | ...... |
| Seitzell, Samuel | ...... | Jersey Shore | * | ...... |
| Seitzinger, Wm. H. | ...... | Wernersville | * | ...... |
| Seltzer, David E. | ...... | Wellsville | * | ...... |
| Seltzer, P. Harvey | 12½ S. 7th st | Lebanon | American Vet. 1885 | Lebanon |
| Senseman, B. F. | 1523 N. 55th st | Philadelphia | * | ...... |
| Sensenich, F. W. | ...... | New Holland | * | Lancaster, 1889 |
| Shaffer, John H. | ...... | Mt. Aetna | * | ...... |
| Shanabrook, Jno. J. | ...... | Blair Station | * | ...... |
| Shanor, Jos., | ...... | Rochester | * | ...... |
| Shaub, Aaron E. | ...... | Lampeter Twp. | * | ...... |
| Shaub, E. K. | Duke & Vine | Lancaster | American Vet., 1890 | Lancaster, Chester, 1890 |
| Shaub, J. K. | ...... | New Holland | * | ...... |
| Shaub, Jno. C. | Duke & Vine | Lancaster | * | ...... |
| Sheffer, John A. | ...... | Codorus Twp. | * | ...... |
| Sheffer, John S. | ...... | Dover Twp. | * | ...... |
| Sheppard, C. W. | 637 Court st | Reading | * | ...... |
| Sherrick, F. N. | 211 E. Apple st | Connellsville | Ontario Vet., 1888 | Fayette, 1889 |
| Sherrick, Harry R. | 211 E. Apple st | Connellsville | Ontario Vet., 1889 | Fayette, 1890 |
| Shields, Wm. A. H. | Ashland Hotel 7th & Race sts | Philadelphia | V. D. Univ. Pa., 1893 | Philadelphia |
| Shiffert, John | ...... | Chambersb'g | * | ...... |
| Shimer, Allen S. | N. Main st | Bangor | Ontario Vet., 1883 | Chester, Lehigh, Northamp., 1891 |
| Shimer, W. S. | 12 Calvert Blk. | Altoona | * | ...... |
| Shimp, Geo. E. | ...... | Ephrata | * | ...... |
| Shink, Abel | ...... | Richfield | * | ...... |
| Shipley, P. H. | ...... | Montgomery Twp. | * | ...... |
| Shoemaker, Harry C. | 1713 N. 12th st | Philadelphia | * | ...... |
| Short, Enoch | ...... | Patton | * | ...... |
| Shue, Franklin F. | ...... | Yorkana | ...... | York, 1895 |
| Shugar, H. B. | ...... | Lebanon | * | ...... |
| Shults, Zebulon | 305 Railroad st | Bloomsburg | * | ...... |
| Sieber, Cyrus | ...... | Mifflintown | * | ...... |
| Sill, J. E. | ...... | Union City | * | ...... |
| Simmons, J. W. | ...... | Silver Spring Twp. | * | ...... |
| Simpson, N. C. | ...... | Mahoning Twp. | * | ...... |
| Single, John L | ...... | Silver Spring Twp. | * | ...... |
| Single, Joseph | ...... | Silver Spring Twp. | * | ...... |
| Sitterly, C. M. | ...... | Scranton | * | ...... |
| Sitterly, J. R. | ...... | Scranton | * | ...... |

| *Name.* | *Street.* | *Town.* | *College, Graduated.* | *County, Registered.* |
|---|---|---|---|---|
| Slaughter, Wm. T. | ..... | Bristol | * | ...... |
| Sloan, Andrew J. | ...... | Dicksonburg | * | ...... |
| Sloan, James M. | Main st | Jamestown | Ontario Vet., 1887 | Mercer, 1889 |
| Sloan, L. D. | ...... | Conneautville | * | ...... |
| Slotzfur, Amos E. | ...... | S. Hermitage | * | ...... |
| Smith, Aloy | ...... | Mt. Carmel | * | ...... |
| Smith, Frank J. | ...... | Bedford | * | ...... |
| Smith, Frank L. | 2150 Mervine st | Philadelphia | V. D. Univ. Pa., 1890 | Philadelphia, 1890 |
| Smith, James B. | ...... | Curwensville | * | ...... |
| Smith, John B. | ...... | Henrietta | * | ...... |
| Smith, John D. | ...... | Dallastown | * | ...... |
| Smith, Jos., | ...... | W. Hanover Twp. | * | ...... |
| Smith, Jos. H. | ...... | Waltersburg | * | ...... |
| Smith, J. M. | ...... | Leatherwood | * | ...... |
| Smith, Morris | ...... | Wellsboro | * | ...... |
| Smith, S. H. | ...... | Piney | * | ...... |
| Smith, T. C. | ...... | Oxford Twp. | * | ...... |
| Snyder, David S. | ...... | Balm | * | ...... |
| Snyder, E. | ...... | Orwigsburg | * | ...... |
| Snyder, J. G. | 208 E. 2d st | Oil City | * | ...... |
| Snyder, John | ...... | Advance | * | ...... |
| Snyder, Noah S. | ...... | Colmar | * | ...... |
| Snyder, O. M. | ...... | Lehighton | * | ...... |
| Snyder, Peter | ...... | Berlin | * | ...... |
| Snyder, S. D. | ...... | Balm | * | ...... |
| Sollenberger, John | ...... | Dillsburg | * | ...... |
| Sonash, John | ...... | Claridge | * | ...... |
| Sowers, John B. | ...... | Chambersb'g | * | ...... |
| Spangler, W. H. | ...... | Rossville | * | ...... |
| Speck, A. Harvey | ...... | Annville | American Vet., 1887 | Lebanon, 1889 |
| Speck, John A. | ...... | Sherman's Dale | * | ...... |
| Spencer, J. J. | ...... | Millerton | * | ...... |
| Spengler, Jared | ...... | Scullhill | * | ...... |
| Spicer, Chas. A. | 1323 Federal st | Allegheny | Ontario Vet., 1889 | Allegheny, Washington, 1890 |
| Spindler, J. Ernest | 702 Duquesne Way | Pittsburg | V. D. Univ. Pa., 1898 | Allegheny, 1901 |
| Spohn, John | ...... | Homestead | * | ...... |
| Stage, Albert F. | ...... | Morris | * | ...... |
| Stambaugh, John | ...... | Codorus Twp. | * | ...... |
| Stambaugh, Nathan | Baltimore st | Hanover | ...... | York, 1889 |
| Stamp, Wm. G. | ...... | Mortonville | * | ...... |
| Standen, Frank | 1220 Locust st | Philadelphia | ...... | Philadelphia, 1889 |
| Stanton, David C. | ...... | Factoryville | ...... | Wyoming, 1889 |
| Stark, G. W. | ...... | East Lemon | * | ...... |
| Stark, T. R. | ...... | Mill City | * | ...... |
| Stauffer, W. B. | 1309 N. 28th st | Philadelphia | * | ...... |
| St. Clair, J. H. | 762 South st | Indiana | ...... | Indiana, 1890 |
| Stecker, John J. | ...... | Brodheads'le | * | ...... |
| Steffy, Henry B. | ...... | E. Coralico | * | ...... |
| Stein, Howard L. | ...... | Friedensburg Oley P. O. | Ontario Vet., 1893 | Berks, 1894 |

